# Advances in Artificial Intelligence for Gastrointestinal Endoscopy: 2026 Update

**DOI:** 10.3390/diagnostics16142248

**Published:** 2026-07-18

**Authors:** Felix Lopez Dominici, Michael B. Wallace

**Affiliations:** Division of Gastroenterology and Hepatology, Mayo Clinic, Jacksonville, FL 32224, USA; lopezdominici.felix@mayo.edu

**Keywords:** clinical diagnostics, medical AI, gastrointestinal endoscopy

## Abstract

Artificial intelligence (AI) is transforming gastrointestinal (GI) endoscopy into a more standardized, data-driven, and workflow-integrated field. Advances in computer-assisted detection (CADe), diagnosis (CADx), quality assessment (CAQ), natural language processing (NLP), and multimodal deep learning have expanded AI applications across colonoscopy, upper endoscopy, endoscopic ultrasound (EUS), ERCP, cholangioscopy, and capsule endoscopy. These systems have demonstrated improvements in lesion detection, procedural quality assessment, workflow efficiency, and diagnostic support. However, current evidence remains largely focused on surrogate outcomes rather than patient-centered clinical benefits, while challenges related to generalizability, explainability, regulatory oversight, automation bias, and workflow integration continue to limit widespread adoption. Future progress will depend on prospective real-world validation, diverse datasets, explainable AI frameworks, and careful integration of human–AI interaction into clinical practice. Overall, AI is evolving from a supportive adjunct into an increasingly integrated component of gastrointestinal endoscopy with the potential to improve procedural quality, diagnostic consistency, and clinical efficiency.

## 1. Introduction

Artificial Intelligence (AI) is rapidly reshaping gastrointestinal (GI) endoscopy into a data-informed and more standardized diagnostic discipline that augments rather than replaces human expertise [1]. By addressing well-recognized limitations in human performance, including operator dependency, interobserver variability, and inconsistencies in procedural performance, AI is emerging as a clinically relevant tool with the potential to advance the field of gastroenterology [2,3,4].

The rapid expansion of the AI literature reflects growing interest in its clinical applications; however, this growth has been uneven across specialties, with imaging-dominant fields accounting for the majority of publications in 2025, while gastroenterology represents a smaller but rapidly evolving domain, as summarized in a recent preprint by Edara et al. [5]. Alongside this growth, there has been a shift in the types of models developed. While large language models (LLMs) remain the most commonly studied approach, recent advances have focused on vision-based and multimodal models that are more directly applicable to endoscopic practice. These developments are particularly relevant to endoscopy, a real-time, visually driven discipline in which clinical performance depends on continuous image interpretation and immediate decision-making. They highlight the need to evaluate AI not only in terms of diagnostic accuracy but also in its ability to integrate into procedural workflows and support real-time clinical decision-making.

Recent expert consensus-based recommendations from the American College of Gastroenterology (ACG), World Endoscopy Organization (WEO) and the American Society for Gastrointestinal Endoscopy (ASGE) formalized the expanding role of AI across endoscopic practice while also addressing ethical, practical, and regulatory considerations. AI is now recognized as an opportunity to enhance patient outcomes through improved detection and characterization of gastrointestinal lesions, more accurate risk stratification, and the standardization and development of procedural quality metrics [1,6,7].

The clinical impact of AI varies across endoscopic modalities and is best understood within each specific clinical context. In this narrative review, we examine recent advances in AI applications directly relevant to gastrointestinal endoscopy, with a focus on image-based detection, characterization, quality assessment, workflow optimization and their clinical implications across the major endoscopic modalities.

## 2. Methods

This narrative review was informed by a structured literature search conducted using PubMed/MEDLINE, Embase, Scopus, and Google Scholar, covering publications available through May 2026. The search strategy was adapted for each database, incorporating free-text keywords combined using Boolean operators. Search terms included combinations of “artificial intelligence,” “machine learning,” “deep learning,” “computer-aided detection,” “computer-aided diagnosis,” “endoscopy,” “colonoscopy,” “upper endoscopy,” “capsule endoscopy,” “cholangioscopy,” “ERCP,” and “EUS.” The reference lists of relevant articles and current society guidelines were also reviewed to identify additional publications. Relevant articles were identified by title, abstract, and full-text assessment and were grouped thematically according to application domains in gastrointestinal endoscopy. Articles were selected based on clinical relevance, with preference given to prospective investigations, multicenter studies, randomized controlled trials, meta-analyses, recent society guideline documents, and landmark studies where appropriate. When multiple studies addressed similar topics, emphasis was placed on more recent publications. Only English-language publications were included. Conference abstracts without full-text publication, duplicate reports, and studies not directly related to gastrointestinal endoscopy were excluded. As a narrative review, the objective is to provide a clinically focused synthesis of key advances in artificial intelligence for gastrointestinal endoscopy rather than a comprehensive systematic review of all available evidence.

## 3. Colonoscopy

Colonoscopy represents the most mature and clinically integrated application of artificial intelligence in gastrointestinal endoscopy, particularly in the domains of lesion detection and characterization. Early evidence raised uncertainty regarding whether computer-assisted detection (CADe) improves detection of clinically significant colorectal neoplasia, particularly advanced lesions, and real-world observational data were similarly cautious [8,9,10].

Nevertheless, CADe systems have consistently demonstrated improvements in adenoma detection rates (ADRs) and reductions in miss rates in both randomized controlled and real-world experience trials [11,12]. More recent randomized controlled trial data in patients with a positive fecal immunochemical test (FIT) in colorectal cancer (CRC) screening further support these findings and suggest that the current value of AI lies primarily in reducing the omission of small lesions, while its impact on clinically significant pathology remains an area of active investigation [13]. Consistent with this, a recent meta-analysis in a FIT-based population demonstrated improved detection of adenomas and sessile lesions with CADe, with limited effect on advanced lesions (≥10 mm, high-grade dysplasia, villous histology) [14]. Similarly, a meta-analysis comparing widely commercialized and locally developed AI-assisted colonoscopy systems demonstrated heterogeneous performance across platforms. While most systems outperformed conventional colonoscopy in several detection metrics, no consistent improvement was observed for high-risk lesions, including sessile serrated lesion detection rate and advanced adenoma detection rate [15].

However, other randomized evidence, including the multicenter, multicountry EAGLE trial conducted in Europe, indicates that CADe may also enhance detection of clinically relevant lesions such as large adenomas and sessile serrated lesions, while supporting scalable, cloud-based, real-time integration into routine practice [16]. This cloud-based architecture further enables continuous deployment of updated algorithms within existing hospital networks, reducing reliance on local hardware and lowering infrastructure requirements. It also allows for seamless integration of additional applications, such as inflammatory bowel disease (IBD) scoring and Barrett’s esophagus neoplasia detection, without the need for on-site engineering support, thereby enhancing scalability and adaptability within routine clinical workflows. This approach may also support training on more diverse datasets, potentially mitigating known biases in AI systems [6,7].

Collectively, these studies support the benefit of CADe for improving adenoma detection and reducing miss rates, but the magnitude of benefit varies across screening settings, baseline endoscopist performance, AI platforms, and study populations. Across randomized trials and meta-analyses, improvements are most consistent for ADRs and diminutive lesions, whereas effects on advanced adenoma, sessile serrated lesions, and other clinically meaningful outcomes are less consistent. In addition, external validation across diverse real-world practice settings remains limited for many commercially available and investigational systems. Future prospective multicenter studies should therefore prioritize clinically meaningful endpoints, including advanced adenoma detection, interval colorectal cancer, and downstream patient-centered outcomes rather than ADRs alone.

### 3.1. AI in Quality Metrics for Colonoscopy

Following the 2024 Quality Task Force recommendations by the ACG and ASGE, recent studies have explored the use of artificial intelligence to quantify procedural quality metrics, including novel withdrawal time measurements that integrate both duration and quality of mucosal inspection [17,18,19,20,21]. AI-based systems enable objective assessment of image quality during withdrawal, allowing for a more refined evaluation of examination quality beyond conventional withdrawal time. Concepts such as Effective Withdrawal Time (EWT) described by Lui et al. and more recently Qualified Mucosal Observation Time (QMOT) described by Li et al. reflect this shift toward quality-adjusted inspection metrics.

Li et al. proposed and prospectively validated an AI system that quantifies QMOT using image quality assessment and anatomical landmark recognition. The system identifies “qualified” frames based on image quality features such as clear mucosal or vascular visualization, adequate illumination, and absence of artifacts. QMOT is then calculated as the proportion of qualified frames relative to total withdrawal frames, multiplied by total withdrawal time. A high QMOT (≥90 s) was associated with increased adenoma detection rates (ADRs) compared with low QMOT (<90 s) (36.45% vs. 19.94%) as well as improved detection of diminutive (OR, 3.93; 95% CI, 2.09–7.39) and small adenomas (OR, 1.76; 95% CI, 1.02–3.03). However, no significant association was observed for advanced adenomas or sessile serrated lesions (*p* = 0.521 and *p* = 0.176, respectively) [17]. Further analysis identified high QMOT as an independent predictor of adenoma detection. These findings suggest that AI-derived quality metrics improve detection of subtle lesions, supporting a transition toward quality-adjusted assessment of mucosal inspection, while their impact on clinically significant pathology remains limited.

Although these AI-derived quality metrics represent an important advance toward more objective assessment of colonoscopy performance, the current evidence is still largely derived from early prospective validation studies. Broader external validation across diverse healthcare systems, endoscopist populations, and endoscopy platforms will be essential before widespread implementation.

### 3.2. AI for Workflow Automation

From a practical perspective, artificial intelligence is likely to benefit physicians across specialties by improving documentation and reporting processes [22,23]. For example, in gastrointestinal endoscopy, particularly colonoscopy, the procedural workflow extends well beyond the examination itself. Current workflows involve pre-procedure evaluation, intra-procedural documentation, lesion detection and characterization, pathology integration, surveillance recommendation generation, and longitudinal follow-up planning. Endoscopists must often retrospectively synthesize information from nursing documentation, technician input, prior medical records, and relevant clinical history while simultaneously documenting procedural findings, interventions, bowel preparation quality, and withdrawal time. In addition, surveillance intervals may initially rely on real-time optical assessment and later require revision once histopathologic results become available.

By automating reporting, enabling real-time data capture, and integrating procedural and electronic health record data, AI-assisted systems may reduce administrative and cognitive burden, improve documentation consistency and workflow efficiency, and allow clinicians to devote greater attention to patient-centered care and clinical decision-making [24].

Ambient AI tools, including natural language processing (NLP)-based systems, offer a complementary approach by automating documentation and reducing cognitive load [6,24]. By capturing and structuring clinical information in real time, these systems may decrease reliance on manual data entry and streamline post-procedural reporting, allowing clinicians to focus more on direct patient care. In an academic outpatient setting, Duggan et al. evaluated an electronic health record (EHR)-integrated ambient scribe system and found that clinicians reported reduced mental fatigue, improved documentation efficiency, and greater engagement with patients. These findings support the potential of ambient AI to enhance both efficiency and the quality of physician–patient interactions.

Similarly, a pilot study from a Canadian center evaluated AIDREA (A.I. VALI Inc., Toronto, ON, Canada), a real-time NLP-based colonoscopy reporting system that supports verbal dictation, image annotation, and capture of procedural data such as withdrawal time [25]. Despite promising results, important limitations remain. The Canadian pilot reported constraints in dictation duration and difficulty processing longer narrative inputs and complex medical terminology, indicating that further refinement and validation are needed. Taken together, this highlights that ambient AI can improve both reporting efficiency and the quality of clinical encounters, while underscoring the need for continued refinement of NLP-based systems and further validation in clinical settings.

**Figure 1:**
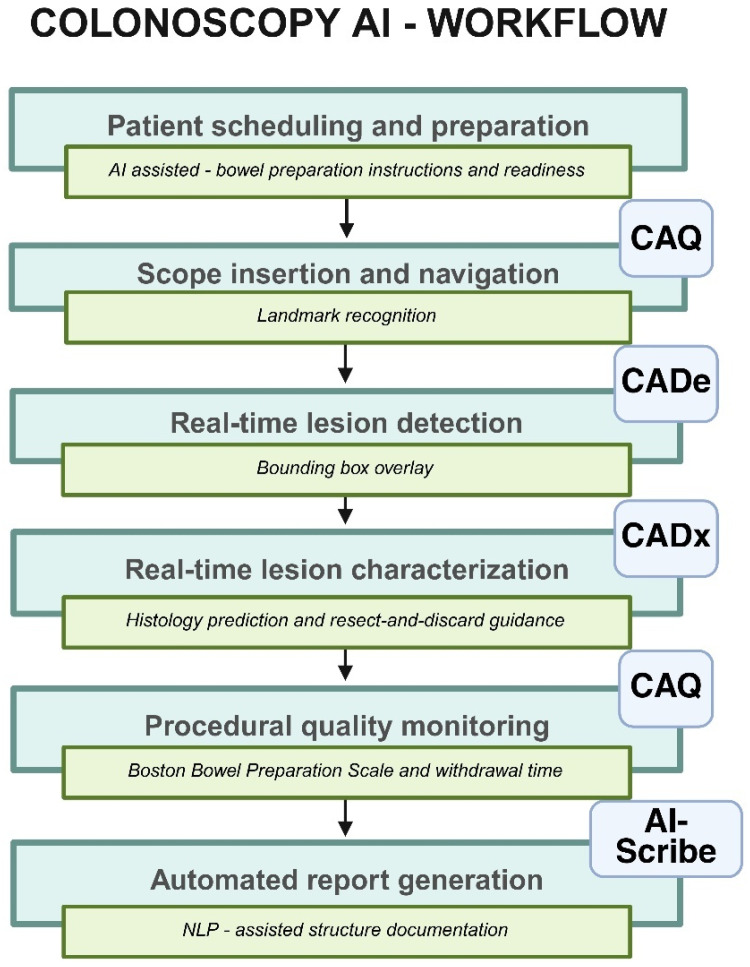
AI-assisted colonoscopy workflow. Sequential roles of CAQ, CADe, CADx, and NLP-based documentation tools across procedural phases. CAQ applications may operate across multiple stages of the procedure, including scope insertion/navigation through landmark recognition and procedural quality monitoring during the examination. CADe, computer-assisted detection; CADx, computer-assisted diagnosis; CAQ, computer-assisted quality; NLP, natural language processing. Black arrows indicate the sequential progression of the colonoscopy workflow. Created in BioRender.com. Lopez Dominici, F. (2026) https://BioRender.com/01nrqqg.

The integration of CADe, CADx, and CAQ systems has extended AI’s role in endoscopy beyond documentation, consolidating detection, diagnostic support, and procedural quality assessment within a single workflow (Figure 1). Platforms such as ColoMaia, ColonPRO, and the cloud-based CADDIE system represent early implementations of this integrated approach, with growing evidence supporting feasibility across diverse clinical settings [26,27,28]. However, the literature is not uniformly positive, and some studies suggest that widespread adoption of AI-based documentation tools may not translate into improved productivity or financial outcomes at the system level [29].

## 4. Upper Endoscopy

Within upper endoscopy, Barrett’s esophagus is a widely studied pathology for AI applications in recent years, particularly within CADe and computer-aided characterization (CADx) [30,31,32,33,34,35,36]. However, current translation into routine clinical practice is yet to be demonstrated, in part due to variability in endoscopic image quality acquisition and regulatory requirements, especially for more niche applications compared to colon polyps.

To address these real-world limitations, Jong et al. and the BONS-AI consortium developed a CADe system incorporating several robust-enhancing training strategies. These included the use of video-based training data as a surrogate for community-level variability, replacement of the ImageNet dataset with the publicly available GastroNet-5M for pretraining, which comprises over 5 million unlabeled endoscopic images, and the use of Vision Transformer (ViT) architecture as an alternative to conventional convolutional neural network (CNN) models such as ResNet-50. Additional data augmentation techniques, including contrast adjustment, image cropping, blurring, and rotation, were also applied to improve generalizability [37].

Consequently, this “robust” CADe system demonstrated improved performance compared with conventionally trained models, achieving area under the curve (AUC) values of 0.92 (*p* = 0.0039), 0.93 (*p* = 0.0006), and 0.85 (*p* = 0.0001) in high-, moderate-, and low-quality test sets, respectively, although some degradation persisted under lower-quality conditions. Secondary analyses identified video-based training and domain-specific pretraining datasets as the primary drivers of these improvements. Importantly, the benefit of video-based training in lower-quality test data was not solely attributable to increased dataset size but rather to greater diversity in image quality, further emphasizing the importance of data diversity for model generalizability and scalability.

Extending beyond technical performance, the increasing development of AI models for surveillance raises important questions regarding their economic impact on healthcare systems. A recent study from Australia evaluated this using a Markov model simulating the natural history of Barrett’s esophagus progression in 1000 individuals, incorporating population-level data from the 2021 national census [38]. The analysis suggested that AI-assisted surveillance may be cost-effective within their healthcare system, although these findings are contingent upon the assumptions underlying the model. Notably, the use of AI was associated with a ≥22% relative increase in detection of high-grade dysplasia and early-stage (T1) lesions, corresponding to a reduction in the incidence of advanced esophageal adenocarcinoma by 3.5% and 1.6% with 3- and 5-year surveillance intervals, respectively. Nevertheless, these projections are based on decision-analytic modeling rather than prospective clinical outcome data. Whether these improvements in lesion detection translate into reductions in esophageal cancer mortality, improved survival, or lower healthcare costs in routine clinical practice remains to be established through long-term prospective studies.

Moreover, image-enhanced technologies combined with artificial intelligence are emerging as a complementary strategy to improve lesion detection during upper endoscopy. In a recent study, Weng et al. evaluated the spectrum-aided visual enhancer (SAVE), which applies hyperspectral enhancement to conventional white-light imaging (WLI), and demonstrated improved diagnostic performance across several YOLO-based computer-aided detection models [39]. The best-performing model achieved higher sensitivity for both esophageal squamous cell carcinoma (81.3% vs. 75.8%) and dysplasia (79.5% vs. 70.4%) compared with conventional WLI alone, suggesting that AI-assisted spectral enhancement may improve early lesion detection. In addition, integration of SAVE significantly improved the F1 score for squamous cell carcinoma (87.0% vs. 83.7%) and dysplasia (80.8% vs. 75.2%), while also increasing the mean average precision (mAP50) for both malignant (86.7% vs. 76.5%) and premalignant (84.1% vs. 68.3%) lesions. Although these findings are promising, they are derived from a relatively small dataset acquired using a single imaging system and therefore require external validation across diverse endoscopy platforms and routine clinical practice before broader implementation. Beyond lesion detection, these approaches suggest a broader shift toward AI-assisted computational enhancement of endoscopic imaging, potentially complementing established image-enhancement techniques. Whether they provide incremental clinical benefit over conventional enhanced imaging modalities remains uncertain and will require prospective validation in routine clinical practice.

## 5. Endoscopic Ultrasound (EUS)

For endoscopic ultrasound (EUS), the current evidence suggests that artificial intelligence can meaningfully enhance the characterization of pancreatic lesions. In particular, AI-based systems have shown the ability to distinguish pancreatic carcinoma (PDAC) from non-carcinomatous pancreatic abnormalities, including intraductal papillary mucinous neoplasms (IPMN) and imaging features associated with chronic pancreatitis [40,41,42]. These findings are important because they point to a potential role for AI in improving diagnostic accuracy in cases where conventional EUS interpretation can be challenging and operator-dependent. In addition, a meta-analysis reported that AI-assisted EUS is a promising approach for identifying gastrointestinal stromal tumors (GISTs) and for estimating malignant potential, suggesting that these tools may contribute not only to diagnosis but also to risk stratification [43]. More recently, Ashida and Kuwahara et al. evaluated a novel AI-assisted rapid on-site evaluation (AI-ROSE) system for cytodiagnosis following endoscopic ultrasound (EUS)-guided fine-needle aspiration (FNA) for pancreatic masses [44]. Their model used transformer-based encoder architecture and was trained on a larger image dataset than earlier studies, strengthening its potential relevance for real-time clinical decision-making.

The authors reported an AUC of 0.93 for AI-ROSE, compared with 0.74 for expert endosonographers, 0.66 for non-experts, and 0.75 for cytotechnologists, demonstrating that the AI system outperformed both cytotechnologists and endosonographers in recognizing Pancreatic Adenocarcinoma (PDAC). Despite a relatively high proportion of benign lesions in the test cohort, the model demonstrated a sensitivity of 89.3%, specificity of 98.1%, and overall accuracy of 95.1%. The reported positive and negative predictive values were 96.3% and 94.4%, respectively; however, these results should be interpreted in the context of an imbalanced dataset and require validation in broader clinical populations. Its compatibility with cloud-based platforms also suggests potential utility in settings with limited access to cytopathology expertise. Further prospective studies are needed to validate the clinical utility of AI-ROSE for both fine-needle aspiration (FNA) and fine-needle biopsy (FNB) [45].

Another emerging application of AI in EUS is the real-time video-based identification of gallbladder (GB) polyps. A recent study from Korea evaluated the performance of three AI models analyzing EUS videos for GB polyp assessment and classification into neoplastic versus non-neoplastic lesions [46]. Among these, the EfficientNetB2 model, trained on approximately 4300 EUS video frames, outperformed the other models, achieving a classification accuracy of 87.9%.

Although this performance exceeds that of models based on transabdominal ultrasonography still images, it was comparable to a prior study by the same group using EUS still images in a larger patient cohort with an accuracy of 89.8%. These findings suggest that video-based AI analysis may extend EUS support beyond lesion detection to selected tasks such as lesion characterization and cytologic assessment. However, the current evidence remains largely limited to early feasibility and validation studies, and whether video-based analysis provides a meaningful clinical advantage over high-quality still images remains uncertain. Prospective multicenter studies will be essential to establish its generalizability, clinical utility, and impact on patient management before routine adoption.

## 6. Endoscopic Retrograde Cholangiopancreatography (ERCP) and Cholangioscopy

Despite comparatively limited development relative to other endoscopic modalities, AI has emerged as a promising experimental tool across ERCP, including prediction of procedural need, automated ampulla identification, assessment of cannulation difficulty and stone extraction, real-time optimization of radiation exposure, and risk stratification for post-ERCP pancreatitis (PEP) [41].

In a recent retrospective multicenter study, Chen and colleagues developed and compared four machine learning models for PEP prediction [47]. The authors identified the 15 most significant variables within each model and found eight variables that were consistent across all four algorithms. Notably, pancreatic guidewire passage, endoscopic papillary balloon dilation time greater than 30 s, and hypercalcemia were among the factors associated with increased risk of PEP. Apart from dilation time, which the authors suggest may be related to their frequent use of smaller balloons, these findings are consistent with prior evidence. Collectively, they support the potential of ML-based models to enhance peri-procedural risk stratification. However, the retrospective design and variable selection methodology warrant cautious interpretation and highlight the need for prospective multicenter validation across diverse patient populations and practice settings before routine clinical implementation.

A study published earlier this year by Zhang et al. reported the development and validation of an AI model designed to estimate the optimal biliary stent length during ERCP for common bile duct strictures [48]. The model demonstrated an accuracy of 97% for stricture identification and approximately 86% for stent length estimation, with a tendency to underestimate stent length. To address this limitation, the authors proposed integrating a multimodal approach combining computed tomography (CT) and magnetic resonance cholangiopancreatography (MRCP) with ERCP to enable three-dimensional (3D) anatomical reconstruction.

In addition, the model’s estimation of stent length is approximately fivefold faster than the conventional guidewire method (0.8 vs. 4.0 s), suggesting potential improvements in procedural efficiency. This was associated with an estimated reduction of 202 mGy·cm^2^ in radiation exposure per case. Although promising, prospective multicenter evidence is needed to determine the clinical impact and cost-effectiveness of this approach.

Given the known limitations of ERCP in distinguishing between malignant and benign biliary strictures, AI applications in cholangioscopy have primarily focused on improving diagnostic accuracy in this setting [49,50]. Saraiva et al. trained and validated a deep learning-based CNN model using approximately 96,000 still-frame images from 164 patients across three centers in Porto, Madrid, and New York to differentiate malignant from benign biliary strictures computing the probability on a frame-by-frame basis. The model achieved a sensitivity of 91.7%, specificity of 94.4%, overall accuracy of 92.9%, and positive and negative predictive values of 94.4% and 91.1% respectively, for differentiating malignant from benign biliary strictures. Furthermore, the model also demonstrated strong performance in recognizing high-risk morphological features such as papillary projections, nodules, masses, and abnormal vessels. For papillary projections, it achieved particularly high specificity (sensitivity, 59.8%; specificity, 97.4%), while detection of masses showed both high sensitivity and specificity (92.8% and 93.5%, respectively). Although sensitivity for papillary projections was modest, the high specificity suggests that AI-assisted identification of these features may improve confidence in recognizing morphologic findings associated with malignant biliary strictures. These findings support the feasibility of AI-assisted morphological characterization during cholangioscopy. However, prospective multicenter studies are needed to determine whether this translates into improved diagnostic decision-making and better patient outcomes in routine clinical practice.

## 7. Capsule Endoscopy

Current evidence supports that AI reduces physicians’ workload while improving efficiency in the interpretation of capsule endoscopy images for small bowel disorders [51,52,53,54]. A recent systematic review and meta-analysis by Dhali et al. evaluated the performance of AI-assisted capsule endoscopy compared with conventional reading by gastroenterologists in the detection of small bowel lesions, including erosions, ulcers, polyps, and tumors [55]. In their analysis, AI systems consistently demonstrated higher sensitivity for lesion detection while significantly reducing reading time relative to conventional interpretation.

More recently, these findings have been supported by prospective evidence. In an international multicenter study, Saraiva et al. developed a deep learning model capable of detecting and differentiating vascular lesions, protruding lesions, ulcers, erosions, and hematic residues during small bowel capsule endoscopy [56]. AI-assisted interpretation achieved a significantly higher lesion detection rate than conventional reading (96.1% vs. 76.3%) while reducing the mean interpretation time to only 203 s per examination. Together, these studies suggest that AI-assisted capsule endoscopy may improve diagnostic performance while substantially reducing reading time.

Beyond AI-assisted image interpretation, recent work has explored AI-assisted computational image enhancement as a complementary strategy for improving endoscopic AI performance. Chou et al. introduced the Spectrum-Aided Vision Enhancer (SAVE), a software-based preprocessing framework that transforms conventional white-light images into simulated hyperspectral and narrow-band-like representations before deep learning analysis [57]. Using the retrospective Kvasir-v2 benchmark dataset, SAVE improved classification of several challenging gastrointestinal lesion classes, particularly polyps, ulcerative colitis, and dyed lesion categories. However, these improvements were not consistent across all deep learning architectures, with the greatest gains in accuracy observed in lightweight models such as AlexNet following SAVE preprocessing compared with conventional white-light imaging (84% vs. 81%, respectively). These findings suggest that software-based spectral enhancement may augment AI-assisted image analysis without requiring dedicated imaging hardware. Importantly, although SAVE was proposed as a computational enhancement strategy with potential applications in wireless capsule endoscopy, its initial validation was performed using the conventional endoscopy Kvasir-v2 benchmark dataset rather than capsule endoscopy images. Consequently, prospective multicenter studies evaluating capsule endoscopy-specific datasets, such as Kvasir-Capsule, that encompass clinically relevant small-bowel lesions will be necessary to determine the clinical applicability of this approach.

While AI-assisted capsule endoscopy consistently improves diagnostic performance and reading efficiency, its effect on outcomes that matter directly to patients remains uncertain. Faster interpretation and higher lesion-detection rates do not necessarily translate into earlier therapy, more frequent changes in management, or lower healthcare costs, because these downstream outcomes have rarely been evaluated [58]. This limitation is evident in the recent systematic review by Al-Juhani et al., which examined studies of obscure small bowel bleeding published over the past decade. Among the eight included studies, none reported patient-level outcomes; instead, most focused on surrogate measures such as reading time or diagnostic yield.

Despite consistent gains in diagnostic efficiency, current evidence has not established that AI-assisted capsule endoscopy translates into meaningful improvements in patient outcomes or clinical decision-making. This represents a critical gap in the current literature. Future prospective studies should evaluate downstream endpoints, including time to diagnosis and treatment, changes in clinical management, resource utilization, cost-effectiveness and patient-centered outcomes.

A summary of the representative primary studies discussed across the major endoscopic modalities is provided in Table 1.

## 8. Future Directions

**Figure 2:**
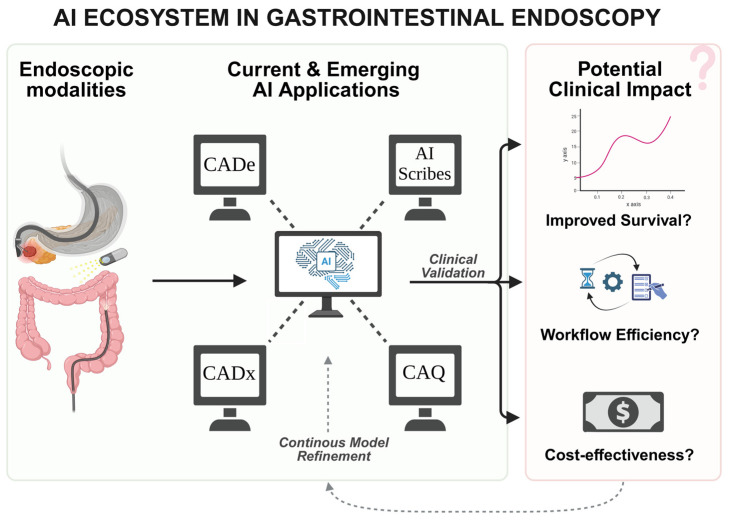
AI ecosystem in gastrointestinal endoscopy. Conceptual overview of current and emerging AI applications across gastrointestinal endoscopy, including computer-assisted detection (CADe), computer-assisted diagnosis (CADx), computer-assisted quality assessment (CAQ), and AI-assisted documentation systems. The figure illustrates the progression from current AI applications through clinical validation toward their potential clinical impact while emphasizing that widespread implementation will require prospective external validation, robust governance frameworks, post-deployment performance monitoring, interoperability with electronic health record systems, and continued human oversight. The anticipated impact of these technologies on clinically meaningful patient outcomes, including improved survival, workflow efficiency, and cost-effectiveness, remains under investigation and requires further prospective evaluation. Solid arrows indicate the primary workflow from endoscopic modalities through AI applications to potential clinical impact. Dashed arrows indicate iterative feedback pathways supporting continuous model refinement as evidence from clinical validation and implementation accumulates. CADe, computer-assisted detection; CADx, computer-assisted diagnosis; CAQ, computer-assisted quality assessment; AI, artificial intelligence. Created in BioRender. Lopez Dominici, F. (2026) https://BioRender.com/lluld6y.

Future work should focus on demonstrating the impact of AI on clinically meaningful outcomes rather than surrogate performance metrics alone. Although improvements in surrogate measures such as adenoma detection rate, sensitivity, specificity, and diagnostic accuracy have consistently supported the value of AI-assisted endoscopy, future prospective studies should determine whether these advances translate into reductions in colorectal cancer incidence and mortality, earlier diagnosis, improved patient management, enhanced quality of life, and lower healthcare-related costs. In addition, further efforts are needed to address key challenges related to scalability, accessibility, equity, generalizability, and cost-effectiveness, including the adoption of cloud-based algorithms and collaborative multi-institutional validation initiatives. These advances represent a step toward a fully integrated AI framework in medicine, encompassing data collection, model development, and effective translation into real-time clinical practice (Figure 2).

As outlined by recent international consensus statements, successful clinical implementation requires robust specialty-society oversight and a clinician-led, staged validation framework to ensure professional accountability and procedural safety [7]. As AI evolves from static diagnostic tools toward adaptive software capable of continuous learning, stakeholders should rigorously manage the AI lifecycle through centralized model registries, version-controlled change logs, and standardized transparency documentation to mitigate model drift and ensure that clinical performance remains stable following software updates [1]. Future implementation strategies should also prioritize seamless interoperability with electronic health record systems, secure data governance through chain-of-custody protocols incorporating robust de-identification and anonymization techniques, and institutional clarity regarding data ownership and stewardship. To ensure equitable deployment, AI algorithms should be validated using heterogeneous datasets that reflect diverse patient populations, thereby improving generalizability and reducing the risk of algorithmic bias [6]. Finally, addressing medicolegal complexities, including the distribution of liability when clinician judgment conflicts with AI recommendations will be essential in defining an evolving standard of care that preserves physician oversight through augmented intelligence rather than autonomous decision-making. Additionally, adherence to AI-specific reporting guidelines, such as TRIPOD-AI for prediction models and STARD-AI for diagnostic accuracy studies, will be important for improving transparency, reproducibility, and the assessment of bias, generalizability, and clinical applicability of future AI systems. This is particularly important because meaningful comparisons across AI applications remain challenging due to differences in study populations, datasets, reference standards, validation strategies, and reported performance metrics. This need has also been highlighted in recent evidence syntheses of AI-assisted endoscopy, which identified heterogeneity in study design and reporting as a major barrier to comparative evaluation [15].

In parallel, model development should increasingly incorporate explainable artificial intelligence (XAI) approaches, such as SHAP and LIME, to improve interpretability and provide insight into the feature-level drivers of model predictions, thereby strengthening physician–AI interaction [59].

A significant challenge in the integration of AI into clinical practice is the risk of physician deskilling, an important concern for both experienced endoscopists and trainees who are still developing foundational diagnostic skills. Prolonged reliance on these systems may foster automation bias, whereby clinicians become overly dependent on algorithmic prompts and may fail to recognize subtle visual findings that AI does not detect. To address this concern, AI should be conceptualized not as a replacement for clinical reasoning but as a scaffold that supports learning transfer and skill development. Future prospective studies are needed to determine whether AI-assisted training and practice preserve independent clinical judgment and core diagnostic competencies when these systems are unavailable [60].

Finally, an important area for further study is patient-centered human–AI interaction, particularly in endoscopy. The successful integration of AI into clinical practice requires careful consideration of the patient perspective, particularly regarding transparency and autonomy [61]. A recent multicenter survey study by Jahagirdar et al. explored patients’ beliefs, familiarity, attitudes, and concerns regarding AI applications in gastroenterology care [62]. A key finding from this study is that most patients emphasized the importance of informed consent prior to any procedure involving the use of AI.

Most patients perceive AI favorably when used as a supportive adjunct rather than as an independent decision-maker, reflecting a strong preference for clinician-led care. Accordingly, healthcare systems must establish transparent frameworks that clearly communicate the role of AI within the diagnostic process. Addressing these patient-centered concerns will be essential for fostering trust and ensuring that technological innovation remains aligned with the ethical principles of human-centered gastrointestinal care.

## 9. Conclusions

The integration of artificial intelligence into gastrointestinal endoscopy marks a major shift in how the field approaches detection, characterization, and procedural quality. In its most established applications, particularly colonoscopy, AI has shown consistent gains in lesion recognition and reductions in physician workload, helping address long-standing challenges such as operator dependence and interobserver variability. These developments are clinically important because they point to a more standardized and reproducible model of endoscopic practice.

At the same time, the role of AI is expanding beyond colorectal screening. Emerging applications in upper endoscopy, endoscopic ultrasound, ERCP, cholangioscopy, and capsule endoscopy suggest that AI may support a broader range of tasks, including risk stratification, procedural optimization, and workflow enhancement. This broader trajectory reflects a field that is moving from proof-of-concept studies toward more practical integration into real-time clinical care.

Despite this progress, important limitations remain. Much of the current evidence still depends on surrogate endpoints such as detection rates, classification accuracy, or procedure time, while data showing direct effects on patient-centered outcomes remain limited. Whether AI can reduce colorectal cancer incidence, improve disease-specific mortality, lower complication rates, or decrease healthcare costs in routine practice remains uncertain. In addition, questions of generalizability, image quality variability, and workflow integration continue to shape the challenge of translating AI systems from controlled study settings into everyday clinical use.

Future progress will depend on more than improved algorithms. It will require prospective real-world validation, representative datasets, and collaboration among clinicians, industry, and regulators to ensure that systems are safe, scalable, and clinically meaningful. Explainable AI will also become increasingly important as models grow more complex, since transparency will matter for trust, auditing, and effective human–AI partnership.

Overall, AI is moving steadily toward becoming an integrated component of endoscopic care rather than a purely supportive technical adjunct. Its long-term value will depend on whether it can improve not only performance metrics but also the outcomes that matter most to patients and clinicians.

## Figures and Tables

**Table 1:** Summary of representative primary studies on artificial intelligence applications across gastrointestinal endoscopic modalities. The table highlights the principal AI application, key findings, major limitations, and corresponding references for each modality discussed in this review. Abbreviations: CADe, computer-assisted detection; CADx, computer-assisted diagnosis; SSL, sessile serrated lesions; PPV, positive predictive value; CRC, colorectal cancer; CAQ, computer-assisted quality assessment; EWT, effective withdrawal time; QMOT, qualified mucosal observation time; ADRs, adenoma detection rate; AI, artificial intelligence; NLP, natural language processing; SAVE, Spectrum-Aided Vision Enhancer; EUS, endoscopic ultrasound; ERCP, endoscopic retrograde cholangiopancreatography; ML, machine learning.

Modality	AI Application	Key Findings	Key Limitations	References
Colonoscopy	CADe + CADx	Improved adenoma/SSL characterization with higher specificity and PPV than conventional colonoscopy.	Single-center study with limited generalizability.	Robles de la Osa et al., 2026 [13].
	Cloud-native CADe	Increased detection of clinically significant lesions while maintaining real-time performance.	Long-term impact on interval CRC remains unknown.	Kader et al., 2025 [16].
	CAQ (EWT/QMOT in separate studies)	AI-derived quality metrics correlated with improved ADRs.	External validation across diverse practice settings is needed.	Lui et al., 2023 [19]; Li et al., 2025 [17].
	Ambient AI/NLP	Improved automated documentation and workflow efficiency.	Performance depends on user engagement and speech recognition accuracy.	Taghiakbari et al., 2025 [25].
Upper Endoscopy	CADe	High diagnostic performance for Barrett’s neoplasia, particularly with high-quality images.	Performance decreases with heterogeneous real-world image quality.	Jong et al., 2025 [37].
	CADe + SAVE	Computational image enhancement improved dysplasia detection.	Evidence is limited to retrospective single-center datasets.	Weng et al., 2025 [39].
Endoscopic Ultrasound	AI-ROSE	High diagnostic accuracy for pancreatic cytologic assessment with rapid interpretation.	Retrospective design and limited validation in inflammatory lesions.	Ashida et al., 2025 [44].
	CADe + CADx	Accurate classification of gallbladder polyps from EUS videos.	Small retrospective cohort requiring prospective validation.	Choi et al., 2025 [46].
Endoscopic Retrograde Cholangiopancreatography	ML risk prediction	Improved prediction of post-ERCP pancreatitis over conventional scores.	Prospective multicenter validation remains necessary.	Chen et al., 2025 [47].
	AI-assisted stent planning (workflow optimization)	Improved stent selection accuracy and reduced radiation exposure.	Limited evaluation in complex biliary strictures.	Zhang et al., 2025 [48].
Cholangioscopy	CADe + CADx	High accuracy for malignant biliary stricture characterization.	Absence of a universal cholangioscopy imaging classification and need for external clinical validation.	Mascarenhas et al., 2025 [50].
Capsule Endoscopy	CADe + CADx	Improved small-bowel lesion detection while reducing reading time.	Clinical impact on patient outcomes remains uncertain.	Mota et al., 2025 [56].
	SAVE	Software-based image enhancement improved AI classification performance.	Validated only on the Kvasir-v2 dataset; prospective validation using capsule endoscopy-specific datasets is needed	Chou et al., 2025 [57].

## Data Availability

No new data were created or analyzed in this study. Data sharing is not applicable to this article.

## Abbreviations

The following abbreviations are used in this manuscript:

AI	Artificial Intelligence
GI	Gastrointestinal
CADe	Computer-assisted detection
CADx	Computer-assisted diagnosis or computer-aided characterization
CAQ	Computer-assisted quality
NLP	Natural language processing
EUS	Endoscopic ultrasound
ERCP	Endoscopic Retrograde Cholangiopancreatography
LLMs	Large language models
ACG	American College of Gastroenterology
WEO	World Endoscopy Organization
ASGE	American Society for Gastrointestinal Endoscopy
ADRs	Adenoma detection rates
FIT	Fecal immunochemical test
CRC	Colorectal cancer
IBD	Inflammatory bowel disease
EWT	Effective Withdrawal Time
QMOT	Qualified Mucosal Observation Time
EHR	Electronic medical record
ViT	Vision Transformer
CNN	Convolutional neural network
AUC	Area under the curve
SAVE	Spectrum-Aided Vision Enhancer
WLI	White light imaging
YOLO	You Only Look Once
SCC	Squamous cell carcinoma
mAP50	Mean average precision
PDAC	Pancreatic adenocarcinoma
IPMN	Intraductal papillary mucinous neoplasms
GISTs	Gastrointestinal stromal tumors
AI-ROSE	AI-assisted rapid on-site evaluation
FNA	Fine-needle aspiration
FNB	Fine-needle biopsy
GB	Gallbladder
PEP	Post-ERCP pancreatitis
ML	Machine learning
CT	Computed tomography
MRCP	Magnetic resonance cholangiopancreatography
3D	Three-dimensional
TRIPOD-AI	Transparent Reporting of a Multivariable Prediction Model for Individual Prognosis or Diagnosis—Artificial Intelligence
STARD-AI	Standards for Reporting Diagnostic Accuracy Studies—Artificial Intelligence
XAI	Explainable artificial intelligence
SHAP	SHapley Additive exPlanations
LIME	Local Interpretable Model-Agnostic Explanations
DL	Deep learning

## References

[1] ASGE, Parasa S., Berzin T., Leggett C., Gross S., Repici A., Ahmad O.F., Chiang A., Coelho-Prabhu N., Cohen J. (2025). Consensus statements on the current landscape of artificial intelligence applications in endoscopy, addressing roadblocks, and advancing artificial intelligence in gastroenterology. Gastrointest. Endosc..

[2] Corley D.A., Jensen C.D., Marks A.R., Zhao W.K., Lee J.K., Doubeni C.A., Zauber A.G., de Boer J., Fireman B.H., Schottinger J.E. (2014). Adenoma detection rate and risk of colorectal cancer and death. N. Engl. J. Med..

[3] Lee T.J., Rees C.J., Blanks R.G., Moss S.M., Nickerson C., Wright K.C., James P.W., McNally R.J., Patnick J., Rutter M.D. (2014). Colonoscopic factors associated with adenoma detection in a national colorectal cancer screening program. Endoscopy.

[4] Sanaka M.R., Deepinder F., Thota P.N., Lopez R., Burke C.A. (2009). Adenomas are detected more often in morning than in afternoon colonoscopy. Am. J. Gastroenterol..

[5] Edara R., Khare A., Atreja A., Awasthi R., Highum B., Hakimzadeh N., Ramachandran S.P., Mishra S., Mahapatra D., Shree S. (2026). Artificial Intelligence in Healthcare: 2025 Year in Review. medRxiv.

[6] Gross S.A., Shaukat A., Afzali A., Ahn J.C., Bajaj J.S., Barkin J.A., Bilal M., Chawla S., Coelho-Prabhu N., Enslin S.M. (2026). Artificial Intelligence for Gastroenterology Practice: A Modified Delphi Consensus. Am. J. Gastroenterol..

[7] Ahmad O.F., Mori Y., Bretthauer M., Dourado D.A., Hassan C., Bisschops R., Bhandari P., Byrne M.F., Dekker E., Mahadevan U. (2026). The Legal and Ethical Framework for Artificial Intelligence in Gastrointestinal Endoscopy: A World Endoscopy Organization International Consensus Statement. Ann. Intern. Med..

[8] Mangas-Sanjuan C. (2023). Role of Artificial Intelligence in Colonoscopy Detection of Advanced Neoplasias. Ann. Intern. Med..

[9] Patel H.K., Mori Y., Hassan C., Rizkala T., Radadiya D.K., Nathani P., Srinivasan S., Misawa M., Maselli R., Antonelli G. (2024). Lack of Effectiveness of Computer Aided Detection for Colorectal Neoplasia: A Systematic Review and Meta-Analysis of Nonrandomized Studies. Clin. Gastroenterol. Hepatol..

[10] Seager A., Sharp L., Neilson L.J., Brand A., Hampton J.S., Lee T.J.W., Evans R., Vale L., Whelpton J., Bestwick N. (2024). Polyp detection with colonoscopy assisted by the GI Genius artificial intelligence endoscopy module compared with standard colonoscopy in routine colonoscopy practice (COLO-DETECT): A multicentre, open-label, parallel-arm, pragmatic randomised controlled trial. Lancet Gastroenterol. Hepatol..

[11] Makar J., Abdelmalak J., Con D., Hafeez B., Garg M. (2025). Use of artificial intelligence improves colonoscopy performance in adenoma detection: A systematic review and meta-analysis. Gastrointest. Endosc..

[12] Xu X., Ba L., Lin L., Song Y., Zhao C., Yao S., Cao H., Chen X., Mu J., Yang L. (2025). Evaluation efficacy and accuracy of a real-time computer-aided polyp detection system during colonoscopy: A prospective, multicentric, randomized, parallel-controlled study trial. Surg. Endosc..

[13] Robles de la Osa D., Santos Fernández J., Pérez Urra C., Espinel Pinedo P., Bulnes Labrador C.B., Martín Ibáñez C., González de Castro E., Pérez Citores L., Montero Moretón Á.M., Santos Santamarta F. (2026). Efficacy of an artificial intelligence system for lesion detection and characterization (CADe and CADx) during colonoscopy following positive faecal immunochemical test in a colorectal cancer screening programme: A randomized clinical trial. Colorectal Dis..

[14] Spadaccini M., Hassan C., Mori Y., Halvorsen N., Gimeno-García A.Z., Nakashima H., Facciorusso A., Patel H.K., Antonelli G., Khalaf K. (2025). Artificial intelligence and colorectal neoplasia detection performances in patients with positive fecal immunochemical test: Meta-analysis and systematic review. Dig. Endosc..

[15] Eman F.N.U., Gyaneshwari F.N.U., Kumari R., Jabeen A., Kumari K., Mansha F.N.U., Sattar S., Zehra F., Hotwani S., Kakar M.T. (2026). Artificial Intelligence–Driven Colonoscopy: A Systematic Review and Network Meta-Analysis on System Performance for Colorectal Neoplasia Detection. JGH Open.

[16] Kader R., Hassan C., Lanas Á., Romańczyk M., Romańczyk T., Kotowski B., Homedes C.S., Mangiavillano B., Bonanno G., Lovat L.B. (2025). A novel cloud-based artificial intelligence for real-time detection of colorectal neoplasia—A randomized controlled trial (EAGLE). npj Digit. Med..

[17] Li W.J., Yan P., Ni M., Zhou J.D., Zhou L., Zhang X., Zhang Z., Gao N., Ji Z., Zhuang D. (2025). An artificial intelligence system for qualified mucosal observation time during colonoscopic withdrawal. npj Digit. Med..

[18] Rex D.K., Anderson J.C., Butterly L.F., Day L.W., Dominitz J.A., Kaltenbach T., Ladabaum U., Levin T.R., Shaukat A., Achkar J.-P. (2024). Quality indicators for colonoscopy. Gastrointest. Endosc..

[19] Lui T.K.L., Ko M.K.L., Liu J.J., Xiao X., Leung W.K. (2024). Artificial intelligence–assisted real-time monitoring of effective withdrawal time during colonoscopy: A novel quality marker of colonoscopy. Gastrointest. Endosc..

[20] Lux T.J., Saßmannshausen Z., Kafetzis I., Sodmann P., Herold K., Sudarevic B., Schmitz R., Zoller W.G., Meining A., Hann A. (2023). Assisted documentation as a new focus for artificial intelligence in endoscopy: The precedent of reliable withdrawal time and image reporting. Endoscopy.

[21] Dimopoulou K., Spinou M., Ioannou A., Nakou E., Zormpas P., Tribonias G. (2025). Artificial intelligence in colonoscopy: Enhancing quality indicators for optimal patient outcomes. World J. Gastroenterol..

[22] Tierney A.A., Gayre G., Hoberman B., Mattern B., Ballesca M., Kipnis P., Liu V., Lee K. (2024). Ambient Artificial Intelligence Scribes to Alleviate the Burden of Clinical Documentation. NEJM Catal..

[23] Bundy H., Gerhart J., Baek S., Connor C.D., Isreal M., Dharod A., Stephens C., Liu T.-L., Hetherington T., Cleveland J. (2024). Can the Administrative Loads of Physicians be Alleviated by AI-Facilitated Clinical Documentation?. J. General. Intern. Med..

[24] Duggan M.J., Gervase J., Schoenbaum A., Hanson W., Howell J.T., Sheinberg M., Johnson K.B. (2025). Clinician Experiences with Ambient Scribe Technology to Assist with Documentation Burden and Efficiency. JAMA Netw. Open.

[25] Taghiakbari M., Wong T., Gaikar R., Azad A., Battat R., Bouin M., Panzini B., Djinbachian R., Armstrong D., von Renteln D. (2025). Exploring a novel voice-guided artificial intelligence platform for real-time colonoscopy documentation: A pilot study. J. Can. Assoc. Gastroenterol..

[26] Medtronic (2026). Medtronic Announces CE Mark for the Next Generation GI Genius™ Module and ColonPRO™ Software. https://news.medtronic.com/Medtronic-announces-CE-Mark-for-the-next-generation-GI-Genius-TM-module-and-ColonPRO-TM-software.

[27] labs M. MAIA LABS. https://maia-labs.com/.

[28] Olympus Corporation of the Americas (2024). First Cloud-Based AI Endoscopy System for Colonoscopy Receives FDA Clearance. https://olympusamerica.com/press-release/2024-09-05/first-cloud-based-ai-endoscopy-system-colonoscopy-receives-fda-clearance.

[29] Liu T.-L., Hetherington T.C., Dharod A., Carroll T., Bundy R., Nguyen H., Bundy H.E., Isreal M., McWilliams A., Cleveland J.A. (2024). Does AI-Powered Clinical Documentation Enhance Clinician Efficiency? A Longitudinal Study. NEJM AI.

[30] Meinikheim M., Mendel R., Palm C., Probst A., Muzalyova A., Scheppach M.W., Nagl S., Schnoy E., Römmele C., Schulz D.A.H. (2024). Influence of artificial intelligence on the diagnostic performance of endoscopists in the assessment of Barrett’s esophagus: A tandem randomized and video trial. Endoscopy.

[31] Hashimoto R., Requa J., Dao T., Ninh A., Tran E., Mai D., Lugo M., El-Hage Chehade N., Chang K.J., Karnes W.E. (2020). Artificial intelligence using convolutional neural networks for real-time detection of early esophageal neoplasia in Barrett’s esophagus (with video). Gastrointest. Endosc..

[32] Jukema J.B., Kusters C.H.J., Jong M.R., Fockens K.N., Boers T., van der Putten J.A., Pouw R.E., Duits L.C., Weusten B.L.A.M., Herrero L.A. (2024). Computer-aided diagnosis improves characterization of Barrett’s neoplasia by general endoscopists (with video). Gastrointest. Endosc..

[33] van der Laan J.J.H., van der Putten J.A., Zhao X., Karrenbeld A., Peters F.T.M., Westerhof J., de With P.H.N., van der Sommen F., Nagengast W.B. (2023). Optical Biopsy of Dysplasia in Barrett’s Oesophagus Assisted by Artificial Intelligence. Cancers.

[34] Hussein M., González-Bueno Puyal J., Lines D., Sehgal V., Toth D., Ahmad O.F., Kader R., Everson M., Lipman G., Fernandez-Sordo J.O. (2022). A new artificial intelligence system successfully detects and localises early neoplasia in Barrett’s esophagus by using convolutional neural networks. United Eur. Gastroenterol. J..

[35] Roser D.A., Ebigbo A. (2025). Future Perspective of Artificial Intelligence Diagnostics for Early Barrett’s Neoplasia. Digestion.

[36] Perananthan V., Konda V.J.A., Leggett C.L. (2026). Narrow Band Imaging and Artificial Intelligence for Detection and Characterization of Barrett’s Esophagus Neoplasia. Gastrointest. Endosc. Clin. N. Am..

[37] Jong M.R., Jaspers T.J.M., Kusters C.H.J., Jukema J.B., van Eijck van Heslinga R.A.H., Fockens K.N., Boers T.G.W., Visser L.S., van der Putten J.A., van der Sommen F. (2025). Challenges in Implementing Endoscopic Artificial Intelligence: The Impact of Real-World Imaging Conditions on Barrett’s Neoplasia Detection. United Eur. Gastroenterol. J..

[38] Tan J.L., Chan J.W.Q., Chinnaratha M.A., Tan K.B., Singh R. (2026). Evaluating the cost-effectiveness of artificial intelligence in Barrett’s surveillance. Endoscopy.

[39] Weng W.-C., Huang C.-W., Su C.-C., Mukundan A., Karmakar R., Chen T.-H., Avhad A.R., Chou C.-K., Wang H.-C. (2025). Optimizing Esophageal Cancer Diagnosis with Computer-Aided Detection by YOLO Models Combined with Hyperspectral Imaging. Diagnostics.

[40] Osagiede O., Wallace M.B. (2025). The Role of Artificial Intelligence for Advanced Endoscopy. Gastrointest. Endosc. Clin. N. Am..

[41] Bharwad A.V., Ahuja R., Jain P., Wadhwa V. (2025). Artificial Intelligence in Pancreatobiliary Endoscopy: Current Advances, Opportunities, and Challenges. J. Clin. Med..

[42] Tacelli M., Lauri G., Tabacelia D., Tieranu C.G., Arcidiacono P.G., Săftoiu A. (2025). Integrating artificial intelligence with endoscopic ultrasound in the early detection of bilio-pancreatic lesions: Current advances and future prospects. Best. Pract. Res. Clin. Gastroenterol..

[43] Zhang B., Zhu F., Li P., Zhu J. (2023). Artificial intelligence-assisted endoscopic ultrasound in the diagnosis of gastrointestinal stromal tumors: A meta-analysis. Surg. Endosc..

[44] Ashida R., Kuwahara T., Koshikawa T., Hashimoto K., Okuno N., Haba S., Kawaji Y., Tamura T., Yamashita Y., Itonaga M. (2026). AI-Assisted Real-Time Cytologic Diagnosis During EUS-FNA of Pancreatic Masses (with Video). Dig. Endosc..

[45] Fujii Y., Matsumoto K., Otsuka M. (2026). A Definite Step Toward Clinical Implementation of AI-Assisted Rapid On-Site Evaluation During EUS-TA. Dig. Endosc..

[46] Choi Y.H., Park J.Y., Lee S.Y., Cho J.H., Kim Y.J., Kim K.G., Jang S.I. (2025). Diagnostic performance of real-time artificial intelligence using deep learning analysis of endoscopic ultrasound videos for gallbladder polypoid lesions. Sci. Rep..

[47] Chen K., Wang L., Wang X., Yang L., Zhang X., Lin Y., Cao L. (2025). Machine learning-derived predictive model for post-ERCP pancreatitis in patients with common bile duct stones: A retrospective multicenter study. Surg. Endosc..

[48] Zhang W.-L., Shao X.-J., Dong X.-Y., Shao H.-T., Li G.-C., Li Z., Zhong N., Ji R. (2026). Artificial intelligence-assisted biliary stent length selection for common bile duct strictures in endoscopic retrograde cholangiopancreatography: Model development and validation. Hepatobiliary Pancreat. Dis. Int..

[49] McCarty T.R., Shah R., Allencherril R.P., Moon N., Njei B. (2025). The Role of Artificial Intelligence Combined with Digital Cholangioscopy for Indeterminant and Malignant Biliary Strictures: A Systematic Review and Meta-analysis. J. Clin. Gastroenterol..

[50] Mascarenhas M., Almeida M.J., González-Haba M., Castillo B.A., Widmer J., Costa A., Fazel Y., Ribeiro T., Mendes F., Martins M. (2025). Artificial intelligence for automatic diagnosis and pleomorphic morphological characterization of malignant biliary strictures using digital cholangioscopy. Sci. Rep..

[51] Brodersen J.B., Jensen M.D., Leenhardt R., Kjeldsen J., Histace A., Knudsen T., Dray X. (2024). Artificial Intelligence-assisted Analysis of Pan-enteric Capsule Endoscopy in Patients with Suspected Crohn’s Disease: A Study on Diagnostic Performance. J. Crohns Colitis.

[52] Spada C., Piccirelli S., Hassan C., Ferrari C., Toth E., González-Suárez B., Keuchel M., McAlindon M., Finta Á., Rosztóczy A. (2024). AI-assisted capsule endoscopy reading in suspected small bowel bleeding: A multicentre prospective study. Lancet Digit. Health.

[53] Bin Y., Peng R., Lee Y., Lee Z., Liu Y. (2025). Artificial intelligence-assisted capsule endoscopy for detecting lesions in Crohn’s disease: A systematic review and meta-analysis. Front. Artif. Intell..

[54] Kwon Y.S., Park T.Y., Kim S.E., Park Y., Lee J.G., Lee S.P., Kim K.O., Jang H.J., Yang Y.J., Cho B.J. (2025). Deep learning-based localization and lesion detection in capsule endoscopy for patients with suspected small-bowel bleeding. World J. Gastroenterol..

[55] Dhali A., Kipkorir V., Maity R., Srichawla B.S., Biswas J., Rathna R.B., Bharadwaj H.R., Ongidi I., Chaudhry T., Morara G. (2025). Artificial Intelligence–Assisted Capsule Endoscopy Versus Conventional Capsule Endoscopy for Detection of Small Bowel Lesions: A Systematic Review and Meta-Analysis. J. Gastroenterol. Hepatol..

[56] Mota J., Rosa B., Mendes F., Mascarenhas Saraiva M., de Moura E.H., Fortes E.B., dos Santos M.E.L., di Palma J., Andrade A.P., Pinto da Costa A. (2025). Real-Life Clinical Validation of Artificial Intelligence-Assisted Detection and Differentiation of Pleomorphic Lesions in Capsule Endoscopy. Am. J. Gastroenterol..

[57] Chou C.-K., Lee K.-H., Karmakar R., Mukundan A., Gade P.C., Gupta D., Su C.-C., Chen T.-H., Ko C.-Y., Wang H.-C. (2025). Emulating Hyperspectral and Narrow-Band Imaging for Deep-Learning-Driven Gastrointestinal Disorder Detection in Wireless Capsule Endoscopy. Bioengineering.

[58] Al-Juhani A., Alzaki A.A., Maghrabi E.F., Rambo R., AlGhamdi A., Alanazi N.A., Desoky R., Desoky M.S. (2025). Artificial Intelligence-Assisted Capsule Endoscopy for Obscure Small-Bowel Bleeding: A Systematic Review of Workflow Gains and the Unmeasured Impact on Patient-Centred Outcomes. Cureus.

[59] Mascarenhas M., Mendes F., Martins M., Ribeiro T., Afonso J., Cardoso P., Ferreira J., Fonseca J., Macedo G. (2025). Explainable AI in Digestive Healthcare and Gastrointestinal Endoscopy. J. Clin. Med..

[60] Budzyń K., Romańczyk M., Kitala D., Kołodziej P., Bugajski M., Adami H.O., Blom J., Buszkiewicz M., Halvorsen N., Hassan C. (2025). Endoscopist deskilling risk after exposure to artificial intelligence in colonoscopy: A multicentre, observational study. Lancet Gastroenterol. Hepatol..

[61] Campion J.R., O’Connor D.B., Lahiff C. (2024). Human-artificial intelligence interaction in gastrointestinal endoscopy. World J. Gastrointest. Endosc..

[62] Jahagirdar V., Srinivasan S., Khalaf K., Siva Mohan Pinnam B., Rama K., Causada Calo N., Desai M., Hayes V., Chhabra R., Campbell J.P. (2026). Patient Perspectives on Artificial Intelligence in Gastroenterology: A Multicenter Survey of Knowledge, Concerns, and Beliefs. Am. J. Gastroenterol..

